# Fracture fragment of the condyle determines the ramus height of the mandible in children with intracapsular condylar fractures treated conservatively

**DOI:** 10.1038/s41598-022-24463-4

**Published:** 2022-11-19

**Authors:** Rui-cong Yang, Meng-juan Cui, Hai-Hua Zhou, Kun Lv, Rong-Tao Yang, Zhi Li, Zu-Bing Li

**Affiliations:** 1 The State Key Laboratory Breeding Base of Basic Science of Stomatology (Hubei-MOST) & Key Laboratory of Oral Biomedicine Ministry of Education, School & Hospital of Stomatology, Wuhan University, Wuhan, People’s Republic of China; 2 Department of Oral and Maxillofacial Surgery, College and Hospital of Stomatology, Wuhan University, 237 Luoyu Road, Wuhan, 430079 Hubei People’s Republic of China

**Keywords:** Paediatric research, Trauma

## Abstract

This study aimed to explore and impart understanding of bone remodelling in children with intracapsular fractures treated conservatively. Records of children (less than 12 years), who sustained intracapsular fractures and treated conservatively, were retrieved consecutively for the period of March 2011 to February 2016. Data about age, gender, date of injury, dates of admission and discharge, mechanism of trauma, location and pattern of fracture, other mandibular fractures, treatment methods and time of review were recorded and analysed. Image dates of pre- and post-treatments, including date of review, were also recorded. A total of 22 patients complete their follow-up and show bone remodelling process. During their follow-up, all the displaced condylar fragments fused with the ramus stump at the displaced position. Regardless of the type of conservative procedure, both treatments cannot promote the spontaneous fracture reduction in patients with intracapsular condylar fractures. During follow-up, the absorption of the lateral process of the condyle after the closed treatment becomes close to the ‘horizontal absorption’, until the height (or articular surface) of the lateral condylar process dropped and aligned to the articular surface of the medial process. In children with intracapsular condylar fractures, the fracture fragment of the condyle determines the ramus height of the mandible. Closed treatment cannot restore the fracture fragment. If the height of the fracture fragments dropped remarkably, then open reduction and rigid internal fixation become more suitable.

## Introduction

Mandibular condyle is a fracture that frequently occurs in younger children. It is of special consequence because the condyle is considered a primary growth centre of the jaw^1,2^. Intracapsular fractures are found predominantly amongst young children and are generally treated by closed treatment. To date, many scholars generally hold the viewpoint that the displaced medial fragment could remodel into its original position even when the medial fragment was notably displaced, and the shortening of the ramus height had been restored according to the capacity for remodelling^3^. However, other scholars denied this view and considered that the reduction of the fracture does not generally occur under closed treatment^4^. However, evidence is insufficient. Our previous research found that the upright position of the fracture fragments of condylar neck or base originates from the skeleton remodelling, rather than the anatomical reduction of the deviated condylar processes^5^.

Therefore, this study aims to explore and impart understanding of bone remodelling in children with intracapsular fractures treated conservatively. We hypothesised that the fracture fragment of the condyle determines the ramus height of the mandible. Present study has found that the ramus height of the mandible is determined by the height of the fracture fragments (or medial process of condyle) in children with intracapsular condylar fractures treated by conservative treatment. In addition, this study indicates that closed treatment cannot restore the fracture fragment in children with intracapsular condylar fractures. If the height of the fracture fragments dropped remarkably, then open reduction and rigid internal fixation become more suitable.

## Patients and methods

From March 2011 to February 2016, 22 patients (less than 12 years) with intracapsular fractures treated conservatively were reviewed retrospectively and consecutively. The institutional review board of Wuhan University approved the protocol, survey and consent forms (approval number: HGGC-146). The study was conducted in accordance with the Helsinki declaration and national regulation on study involving humans. Informed consent was obtained from the legal guardian(s) of all children. Records of children (less than 12 years)^6,7^ who sustained intracapsular fractures and treated conservatively, were retrieved consecutively for the period of March 2011 to February 2016. Data about age, gender, date of injury, dates of admission and discharge, mechanism of trauma, location and pattern of fracture, other mandibular fracture, treatment methods and time of review were recorded and analysed. Image dates of pre- and post-treatment, including date of review, were also recorded. Patients or files were excluded as study subjects based on the following: (1) incomplete information (especially the radiographic data), (2) lack of follow-up data (especially the radiographic data) and (3) intracapsular condylar fractures treated surgically.

The condylar head fractures (intracapsular fractures) were divided into three portions, namely, lateral third (type A), central third (type B), medial third fractures (type C) and comminuted fracture of condylar head (type M), as proposed by He et al.^8^. Patients with type A/B/C fractures were included in the present study, whereas type M fractures were excluded because the ramus height of the mandible decreased seriously during injury.

Conservative treatment of intracapsular condylar fractures was indicated in present cases, as follows: (1) children less than 12 years, (2) intracapsular condylar fracture was not treated surgically before and/or not treated previously in other hospital, (3) new fracture less than 3 weeks. Conservative treatment included occlusal splint combined with maxillo-mandibular traction (achieved by self-drilling cortical bone screws) for 4 weeks, patients were asked to eat fluid diet and do functional training (practice active mouth-opening exercises) in this period. After 4 weeks, the occlusal splint and screws were removed.

Based on the exclusion criteria, 22 patients have completed their follow-up and have shown bone remodelling process.

### Statistical analyses

Descriptive analysis was performed with the SPSS software (version 19.0; SPSS, Chicago, IL). The continuous variables were reported as mean ± SD.

## Results

In the five-year records retrieved during this study, 22 young patients were found to have sustained intracapsular condylar fractures. Amongst them, 14 were boys and eight were girls with a boy/girl ratio of 1.75:1; 15 children were unilateral and seven were bilateral. Patients with intracapsular condylar fractures ranged from 2.4 to 11 years old, with a mean age of 6.68 ± 2.36 years. The shortest time of absorption in computed tomography scan, observed in 31st day and the longest time in 415th day (average time of 112.68 ± 82.95 days). Fall-related accidents were the most common mechanism of injury (14 patients, 63.6%), followed by motor vehicle and motorcycle accidents (five patients, 22.7%). Table 1 shows the details of young patients with intracapsular condylar fractures treated by conservative procedures. During their follow-up, all displaced condylar fragments fused with the ramus stump at the displaced position. Regardless of the type of conservative procedure, both treatments cannot promote the spontaneous fracture reduction in patients with intracapsular condylar fractures. During follow-up, the absorption of the lateral process of the condyle after the closed treatment was close to the ‘horizontal absorption’ until the height (or articular surface) of the lateral condylar process dropped and aligned to the articular surface of the medial process. Sometimes, the simultaneous occurrence of the abduction of condylar head and the displacement of the medial process (fracture fragments) inferiorly lead to the mixed vertical and horizontal absorption. No children patients presented ankylosis of temporomandibular joint during their follow-up.

**Table 1:** Characteristics of the young patients sustained with intracapsular fractures treated conservatively.

Patients	Gender	Age (years)	Etiology	Intracapsular fractures (bilateral/unilateral)	Horizontal absorption (yes/no)	Time of observing absorption (days)
1	Boy	2.4	Fall high	Bilateral	Yes	175
2	Girl	3	Fall high	Unilateral	Yes	56
3	Boy	4	Other	Unilateral	Yes	44
4	Boy	5	Fall groud	Unilateral	Yes	183
5	Boy	5.3	Fall groud	Unilateral	Yes	37
6	Girl	5.3	MVA	Unilateral	Yes	162
7	Boy	5.5	Fall from stair	Bilateral	Yes	31
8	Boy	5.5	Fall groud	Bilateral	Yes	91
9	Girl	5.7	Fall high	Unilateral	Yes	106
10	Girl	6	Fall high	Unilateral	Yes	87
11	Boy	6.1	Fall from stair	Bilateral	Yes	81
12	Boy	6.4	Fall groud	Bilateral	Yes	160
13	Boy	6.6	MVA	Bilateral	Yes	155
14	Boy	7	Fall groud	Unilateral	Yes	95
15	Girl	7	Fall high	Unilateral	Yes	146
16	Boy	7.1	Motorcycle	Unilateral	Yes	49
17	Boy	8	Fall groud	Unilateral	Yes	49
18	Boy	9	MVA	Unilateral	Yes	38
19	Girl	10	Other	Unilateral	Yes	111
20	Girl	10	Fall groud	Unilateral	Yes	100
21	Girl	11	Bicycle	Bilateral	Yes	108
22	Boy	11	Motorcycle	Unilateral	Yes	415

*MVA* motor vehicle accidents.

**Figure 1:**
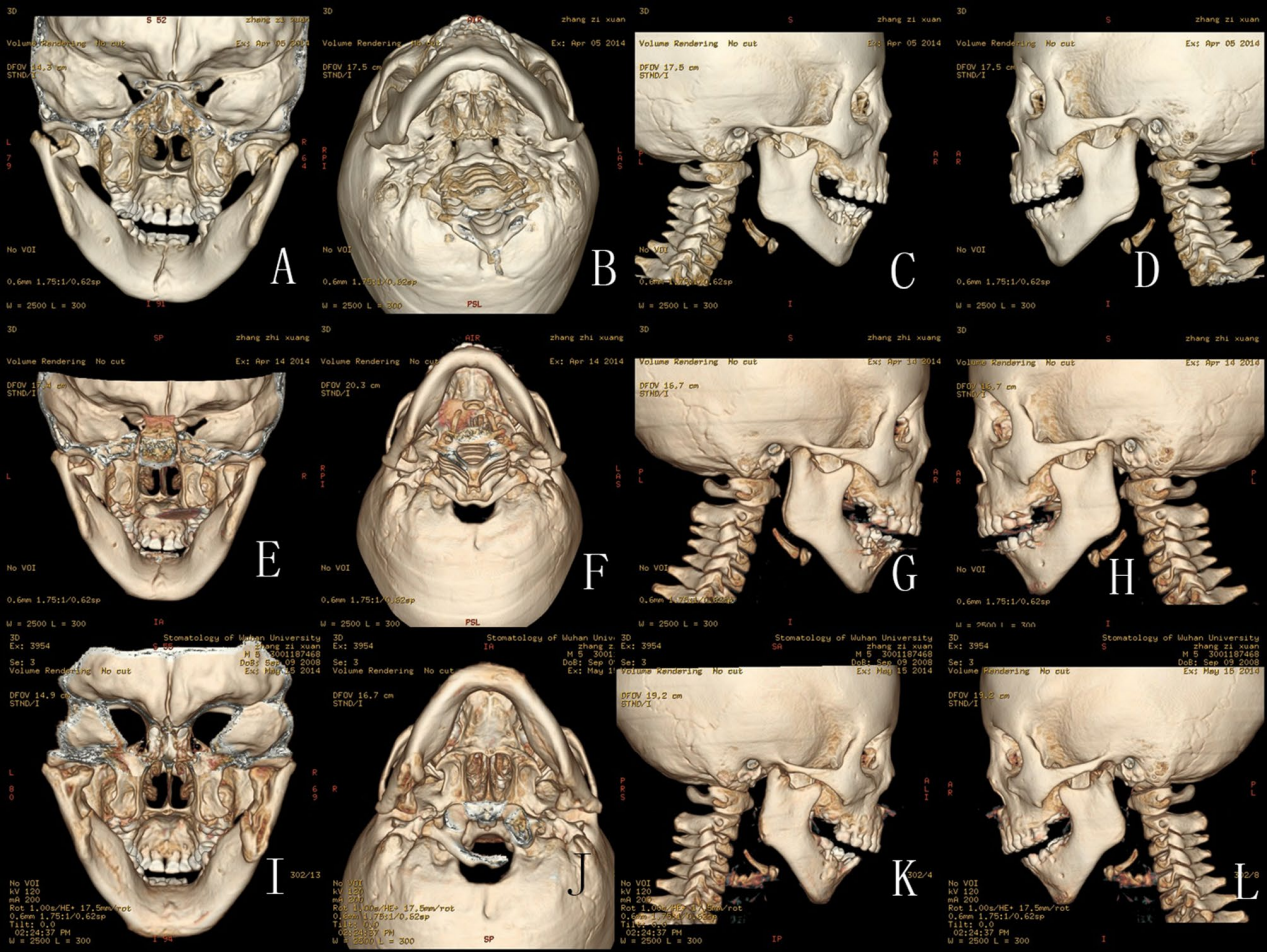
(**A**–**D**) CT scan in April 5th 2014 of a 5.5 year-old boy: (**A**) Back view of the fractures: CT scan showing bilateral intracapsular fractures, and fracture of the middle symphysis; (**B**) Bottom view of the fractures; (**C**) Lateral view of the right condyle; (**D**) Lateral view of the left condyle; (**E**–**H**) CT scan in April 14th 2014 after the treatment: the fracture fragments are still in their previous displaced position; (**I**–**L**) CT scan in May 15th 2014: (**I**) Fracture fragments fused with ramus stump at their displaced position; (**J**) Bottom view of the fractures; (**K**) Lateral view of the right condyle: the lateral head of the condyle resorbed, leaving a spur on the lateral side, the height of ascending ramus on the right decreased obviously; (**L**) Lateral view of the left condyle: the remodelling of left condyle similar to the right side.

**Figure 2:**
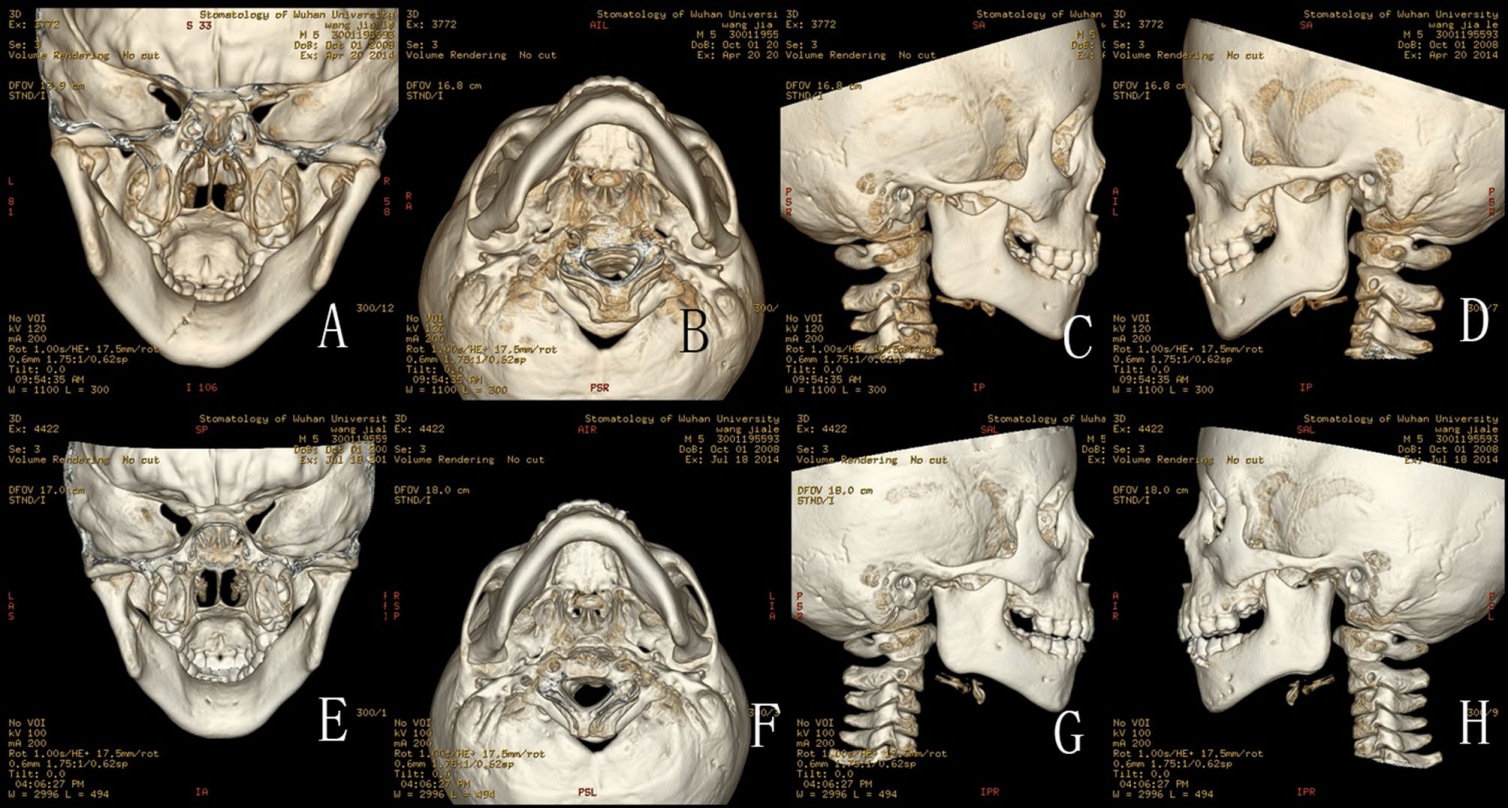
(**A**–**D**) CT scan in April 20th 2014 of a 5.5 year-old boy: (**A**) Back view of the fractures: CT scan showing bilateral intracapsular fractures, and fracture of the left symphysis; (**B**) Bottom view of the fractures; (**C**) Lateral view of the right condyle; (**D**) Lateral view of the left condyle; (**E**–**H**) CT scan in July 18th 2014: (**E**) Back view of the fractures: CT scan showing flat condylar heads with a central notching, fracture fragment fused with ramus stump at their displaced position, the lateral head of the condyle resorbed significantly; (**F**) Bottom view of the fractures; (**G**) Lateral view of the right condyle: the lateral head of the condyle almost resorbed completely, and the height of ascending ramus on the right decreased significantly; (**H**) Lateral view of the left condyle: the remodelling of left condyle similar to the right side.

**Figure 3:**
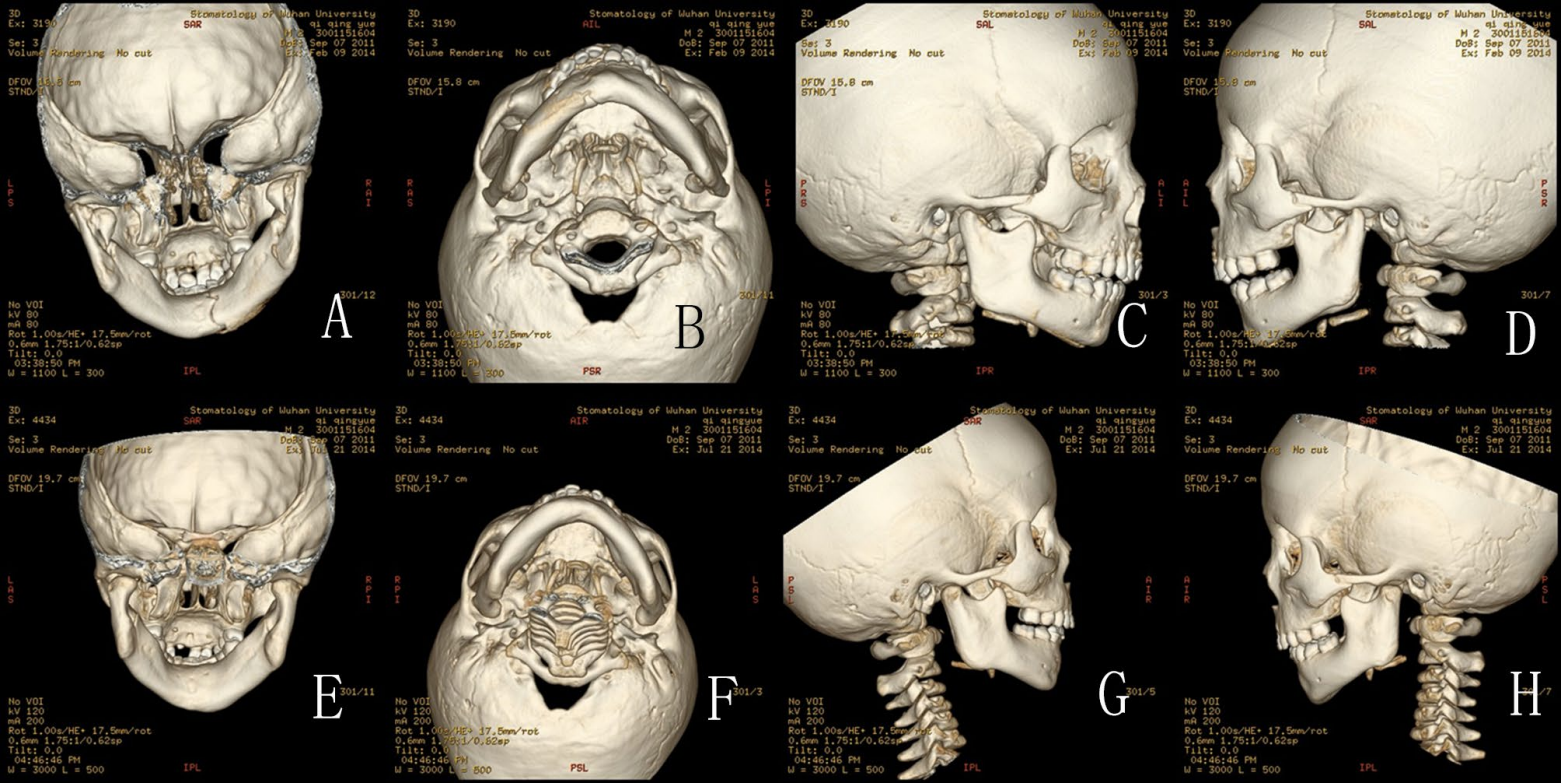
(**A**–**D**) CT scan in February 9th 2014 of 2.4 year-old boys: (**A**) Back view of the fractures: CT scan showing bilateral intracapsular fractures, and fracture of the right symphysis; (**B**) Bottom view of the fractures; (**C**) Lateral view of the right condyle; (**D**) Lateral view of the left condyle; (**E**–**H**) CT scan in July 21st 2014: (**E**) The remoulded condylar heads appeared smooth, however, the lateral heads of the condyle resorbed, leaving spur on the lateral sides; (**F**) Bottom view of the fractures; (**G**) Lateral view of the right condyle: the lateral head of the condyle resorbed obviously, and the height of ascending ramus on the right decreased obviously; (**H**) Lateral view of the left condyle: the remodelling of left condyle similar to the right side.

**Figure 4:**
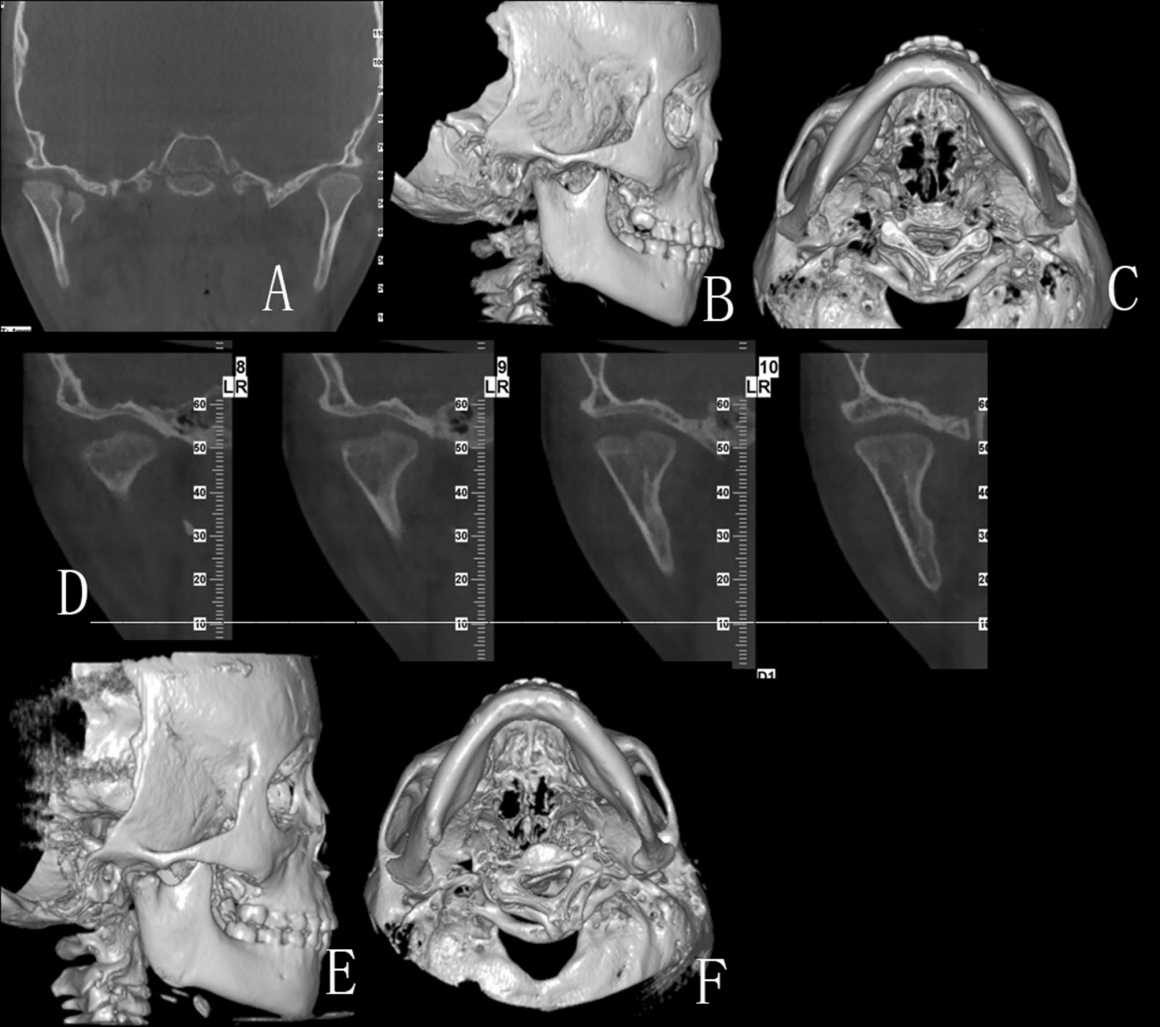
(**A**–**C**) CT scan in September 10th 2012 of a 5 year-old boy: (**A**) Back view of the fractures: CT scan showing right intracapsular fractures; (**B**) Lateral view of the right condyle; (**C**) Bottom view of the fracture; (**D**–**F**) CT scan in March 4th 2013: (**D**) Coronal CT scan showing flat right condylar head with a central notching, fracture fragment fused with ramus stump at their displaced position, the lateral head of the condyle resorbed significantly; (**E**) Lateral view of the right condyle: the lateral head of the condyle resorbed significantly, and the height of ascending ramus on the right decreased significantly; (**F**) Bottom view of the fracture.

**Figure 5:**
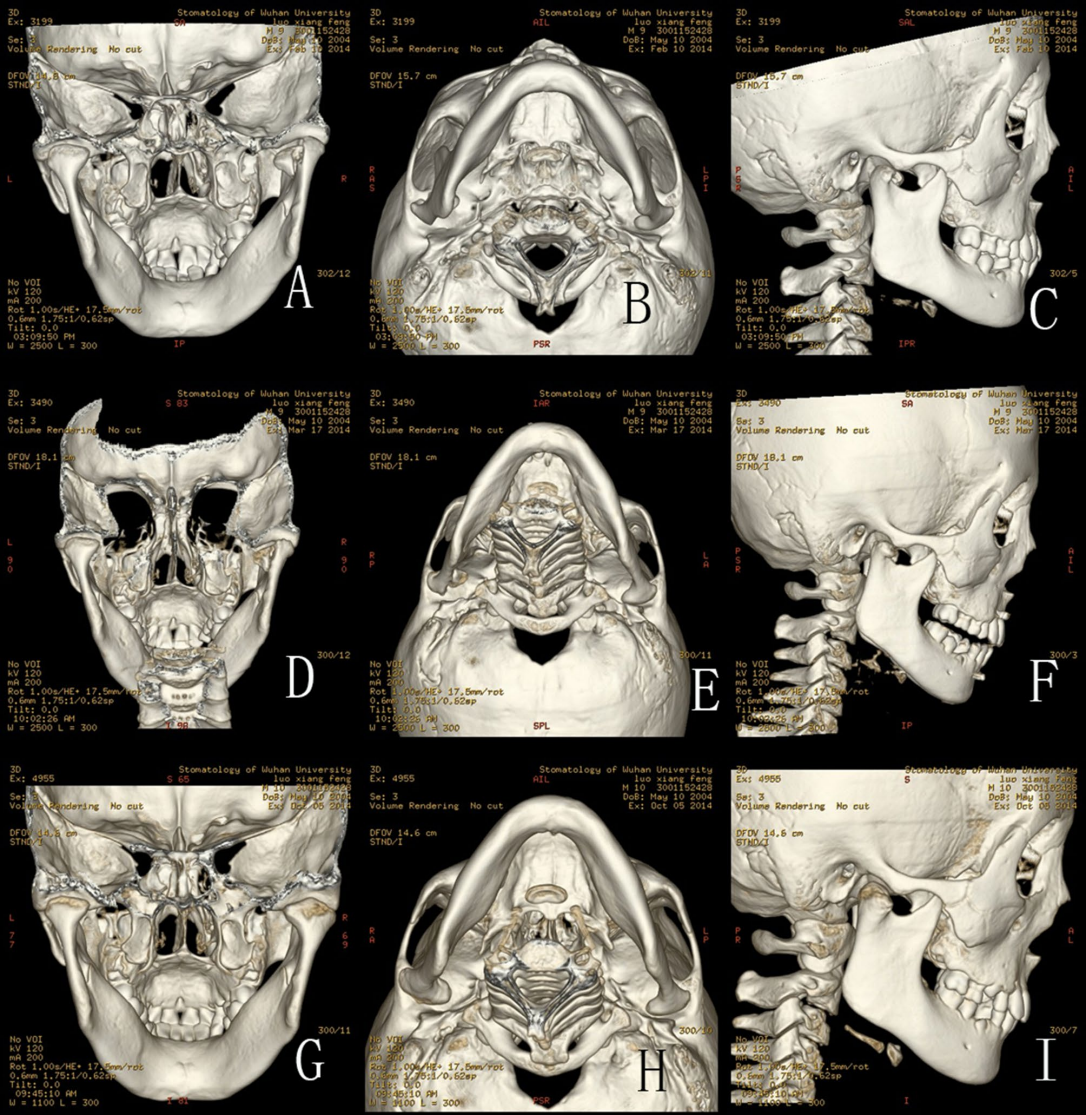
(**A**–**C**) CT scan in February 10th 2014 of a 9 year-old boy: (**A**) Back view of the fractures: CT scan showing right intracapsular fractures; (**B**) Bottom view of the fracture; (**C**) Lateral view of the right condyle; (**D**–**F**) CT scan in March 17th 2014: (**D**) Back view of the fracture: CT scan showing fracture fragment fused with ramus stump at the displaced position; (**E**) Bottom view of the fracture; (**F**) Lateral view of the right condyle: the lateral head of the condyle began to show signs of absorption; (**G**–**I**) CT scan in October 5th 2014: (**G**) Back view of the fractures: CT scan showing the remoulded condylar heads appeared smooth; (**H**) Bottom view of the fracture; (**I**) Lateral view of the right condyle: the lateral head of the condyle resorbed obviously.

Figures 1, 2, 3, 4, 5 show the detailed bone remodelling.

## Discussion

Intracapsular condylar fractures are found predominantly amongst young children and are generally treated by conservative treatment. To date, many scholars generally hold the viewpoint that the displaced medial fragment could remodel into its original position even when the medial fragment was notably displaced, and the shortening of the ramus height had been restored according to the remodelling capacity. This study found that the use of any conservative method to promote spontaneous fracture reduction in patients with intracapsular condylar fractures is impossible. During follow-up, the absorption of the lateral process of the condyle after the conservative treatment was close to the ‘horizontal absorption’, until the height (or articular surface) of the lateral condylar process dropped and aligned to the articular surface of the medial process. These findings have an important impact on clinical practice. This discovery reminds us that the ramus height of the mandible is determined by the height of the fracture fragments (or medial process of condyle) in children with intracapsular condylar fractures treated by closed treatment. If the height of the fracture fragments dropped remarkably, then open reduction and rigid internal fixation may be more suitable. Therefore, further researches are needed in the future.

After the occurrence of intracapsular condylar fractures, the continued traction of the lateral pterygoid muscle results in the anteromedial displacement of the fragment in condylar head fractures^9^. A previous study^10^ revealed that immediately after injury, nearly all intracapsular condylar fractures showed anteromedial displacement of the disc and fractured condylar fragment. Even at 3 months after injury, all patients continued to exhibit displacement of the disc and the condylar segments. In our previous study, we found that the upright position of the extracapsular condylar fragments originates from the remodelling of the skeleton rather than the anatomical reduction of the deviated condylar processes^5^.

This study observed that the use of occlusal splint (or any other conservative methods) to promote the spontaneous fracture reduction in patients with intracapsular condylar fractures is impossible because the horizontal traction force of the lateral pterygoid muscle cannot be eliminated under those circumstances. Ellis and Throckmorton^4^ do not use the term ‘closed reduction’, which they believe is a misnomer, because reduction of the fracture dose not generally occur spontaneously. Nonetheless, the removable occlusal splint^11,12^ is widely used because it is easy to fabricate and comfortable for children to wear; it helps re-establish normal occlusion and allows the mandible to maintain appropriate relationship with the maxilla. In addition, it allows early mobilisation, eating and mandibular exercises and promote haematoma resolution and tissue recovery^12^. They^12^ even stated that wearing the occlusal splint followed by regular exercises resulted in good mandibular function and condylar remodelling in children patients; no patient had TMJ symptoms and ankylosis.

In the past, most previous studies claimed successful remodelling once the condyle head was reconstructed into an arc (or oval) shape. Thorén^13^ found that incomplete remodelling with a flattened or irregular surface of the condylar head associated with deformation of the condylar neck was frequently observed after condylar fracture in childhood. However, some authors had observed that the fractured fragments resorbed completely but with acceptable condylar remodelling^14^. Other authors^12^ showed that in children with unilateral fractures, the condyles were incompletely remodelled with relatively short and flattened condylar heads and flattened glenoid fossa compared with the contralateral normal condyles; most of their patients (children) showed slight difference in length between the fractured and contralateral ramus. Some patients showed condylar deformity^15^ and altered mandibular growth^16^. However, they were usually only judged by visual observation. Previous studies cannot easily provide direct evidence to answer whether or not condylar head resorption exists. This study presents the detailed process of condylar head resorption, but more studies are needed in the future.

The horizontal absorption of the lateral process of the condyle after the closed treatment is surprising. The absorption of the residual condylar head is different from the resorption of the lateral condylar head because of the abduction of the condylar process^17^. Abduction leads to the ‘vertical absorption’ until condylar head located at the concentric position of the glenoid fossa. In the present study, the absorption of the lateral process of the condyle after the closed treatment was close to the ‘horizontal absorption’, until the height (or articular surface) of the lateral condylar process dropped and aligned to the articular surface of the medial process. Sometimes, the simultaneous occurrence of the abduction of condylar head and the displacement of the medial process (fracture fragments) inferiorly lead to the mixed vertical and horizontal absorption. Surprisingly, this absorption only occurs in children and is rarely found in adults in the above situation.

He et al.^18^ indicated that the combination of an intracapsular fracture with concomitant widening of the mandible caused the lateral pole of the condyle or the condylar stump to become displaced laterally or superolaterally in relation to the zygomatic arch, where it fused and formed the TMJ ankylosis. Chang et al.^3^ found that three of the 23 children developed TMJ ankylosis due to ramus stumps displaced laterally and made contact with the root of the zygoma. In the present study, no TMJ ankylosis was found. The important reason is that none of our patients’ condylar stump was displaced laterally or superolaterally to the zygomatic arch post-trauma or after intervention. Clinically over the past decades, we rarely found the occurrence of TMJ ankylosis in children with intracapsular condylar fractures in our department. Zhao et al.^12^ also found no patient (40 children with condylar fractures, most children patients suffered high-neck fractures and intracapsular fractures) had TMJ symptoms and ankylosis. Therefore, we also speculate that the presence of articular cartilage in children prevented them from developing TMJ ankylosis, in spite of the disc displaced anteromedially; whereas in adult, articular cartilage of condyle is generally degenerated. However in present study, we didn’t use Magnetic Resonance Imaging (MRI). Therefore, we can’t assess disc displacement.

Some limitations could be found in this study. First, it is a retrospective study with small sample size. The small size reduced the power but provided the discovery unreported previously. Second, the cases were only obtained from our own hospital (maxillofacial trauma service), some paediatric patients could be brought for care in other children’s hospital, and multicentre and more sample studies are necessary in the future. However, our department was amongst the largest centres for patients with facial trauma in central China, and the children patients were treated consecutively with nearly no omission. Thus, we consider our findings similar to those other large maxillofacial urban units in China.

In conclusion, in children with intracapsular condylar fractures, the fracture fragment of the condyle determines the ramus height of the mandible. Closed treatment cannot restore the fracture fragment. If the height of the fracture fragments dropped remarkably, then open reduction and rigid internal fixation become more suitable.
